# Two‐dimensional robot‐assisted physeal‐sparing medial patellofemoral ligament reconstruction can achieve favourable clinical outcomes for skeletally immature patients with recurrent patellar dislocation

**DOI:** 10.1002/jeo2.70294

**Published:** 2025-07-18

**Authors:** Qiuzhen Liang, Zandong Zhao, Hongwei Zhan, Peidong Liu, Chaofan Liao, Junxuran Li, Yongchao Duan, Xin Kang, Bin Tian, Bo Ren, Jiang Zheng, Liang Zhang

**Affiliations:** ^1^ Sports Medicine Center, Honghui Hospital Xi'an Jiaotong University Xi'an Shaanxi China; ^2^ Department of Imaging The Hospital of Xidian Group Xi'an Shaanxi China; ^3^ Department of Digital Imaging, Honghui Hospital Xi'an Jiaotong University Xi'an Shaanxi China

**Keywords:** MPFL, physeal‐sparing, recurrent patellar dislocation, robot‐assisted

## Abstract

**Purpose:**

The surgical treatment of recurrent patellar dislocation (PRD) in adolescents faces particular difficulties, since the integrity of open growth plates may be compromised by standard surgical methods used in adults. This study aimed to review a series of adolescents with RPD who underwent robot‐assisted physeal‐sparing medial patellofemoral ligament (MPFL) reconstruction, compare the clinical results with those of a non‐robot‐assisted group, and measure the vertical distance between Schöttle point and the physis intraoperatively.

**Methods:**

This retrospective clinical analysis included 55 adolescents with RPD who had no significant bone deformities and underwent MPFL reconstruction using either a robot‐assisted technique or a non‐robot‐assisted method between February 2019 to November 2023. Using a 2D intraopertive navigation system, the vertical distance between Schöttle point and the medial distal femoral physis was measured in the robot‐assisted group. The operation duration, the number of fluoroscopies and guide needle punctures were recorded in both groups. The anterior and distal tilt angles of the bone tunnel, as well as the distance between Schöttle point and the femoral insertion of the bone tunnel (DST), were measured using postoperative CT imaging in both groups. In addition to CT, MRI, and radiographic evaluations, the International Knee Documentation Committee (IKDC), Lysholm and Kujala scores were used to assess the clinical outcomes.

**Results:**

The mean patient age was 13.1 years (range, 11–16 years). At a mean of 35.5 ± 8.5 months postoperativel, all patients returned for evaluation. In the robot‐assisted group, the mean distance from the Schöttle point to medial femoral physis was 6.97 ± 1.92 mm, with all Schöttle points positioned distal to the physis in every case. The IKDC, Lysholm and Kujala scores in the robot‐assisted group were significantly higher than those in the non‐robot‐assisted group three months post‐operatively (87.1 ± 6.1 vs. 82.9 ± 5.7, *p* = 0.011; 85.3 ± 5.7 vs. 81.1 ± 5.2, *p* = 0.007; 82.7 ± 6.0 vs. 77.5 ± 5.1, *p* = 0.001); however, at the last follow‐up, there was no significant difference (*p* > 0.05). No patients experienced recurrent patellar instability or physeal invasion following surgery, and significantly improved functional scores and patellar tilt angles were noted at the final follow‐up (*p* < 0.05). In the robot‐assisted group, the number of fluoroscopy and guide needle punctures was significantly lower (3.7 ± 0.5 vs. 10.3 ± 1.8; 1.1 ± 0.3 vs. 5.7 ± 1.1, *p* < 0.001), with smaller anterior tilt angles (14.5 ± 1.7 vs. 16.6 ± 4.7, *p* = 0.044), larger distal tilt angles (13.8 ± 1.7 vs. 11.4 ± 1.5, *p* < 0.001) and shorter DST (2.00 ± 0.84 vs. 5.45 ± 1.74, *p* < 0.001) compared to the non‐robot‐assisted group.

**Conclusion:**

An anterodistal oblique bone tunnel can be safely used for anatomical MPFL reconstruction in skeletally immature patients, yielding good short‐term clinical outcomes. The robot‐assisted method is more accurate than the freehand method, requiring fewer intraoperative fluoroscopies and enabling faster early recovery.

**Level of Evidence:**

Level IV.

**Abbreviations**
AA: anterior tilt angle of the bone tunnel
DA: distal tilt angle of the bone tunnel
DST: the distance between the Schöttle point and femoral tunnel insertion
IKDC: International Knee Documentation Committee
MPFL: medial patellofemoral ligament
PTA: patellar tilt angle
RPD: recurrent patellar dislocation
TT‐TG: tibial tubercle‐trochlear groove

## INTRODUCTION

The incidence of patellar dislocation ranges from 29 to 40 per 100,000 individuals, with the peak occurrence of first‐time dislocations observed at age 15 [6]. Adolescents presenting with RPD face particular difficulties when it comes to surgery, since the integrity of open growth plates may be comp romised by standard surgical methods used in adults [1]. For patients without significant bone deformities, isolated MPFL reconstruction remains the most widely used and clinically successful surgical approach [3].

Soft tissue procedures were historically considered the preferred surgical approach due to their reduced risk of physeal injury [4, 10, 20]. However, these suture‐based soft tissue fixation techniques may be less reliable than bone tunnel fixation. Additionally, anatomical MPFL repair proves challenging when using soft tissue procedures alone. Anatomical MPFL reconstruction is recommended for skeletally immatuer patients, given the high failure rates associated with traditional surgical techniques, including lateral release, primary repair, medial retinacular imbrication, and the Roux‐Goldthwait procedure in pediatric populations [12].

The femoral insertion of the MPFL is typically located within a 5 mm area currently defined as Schöttle point [16]. Despite persistent debate, most published studies describe the MPFL footprint in skeletally immature patients as located at or distal to the physis [12, 18]. In the present study, Schöttle's point [16] was employed to identify the MPFL insertion and its relationship to the physis. The traditional freehand method involves identifying the Schöttle point on a true lateral view and then the guide pin is positioned to avoid the physis, intercondylar notch and articular cartilage. However, simply placing the bone tunnel more distally and anteriorly fails to sufficiently mitigate physeal injury risk due to significant anatomical variability in the distal femoral physis [5]. Furthermore, it is crucial to emphasize that even minimum variations in the insertion point on the lateral radiographs might cause the exit needle to be significantly malpositioned on the anteroposterior views [5].

Currently, these issues are being addressed more safely and dependably by the creation and clinical use of orthopaedic robots. The aim of this study was to review a series of adolescent patients with RPD who underwent robot‐assisted physeal‐sparing MPFL reconstruction, comparing the clinical outcomes with those of a non‐robot‐assisted group, and to measure the distance between the Schöttle point and the physis intraoperatively. The hypothesis of the study was that robot‐assisted MPFL reconstruction provides a safer, more reliable, and more accurate approach to personalized anatomical reconstruction compared to non‐robotic methods.

## METHODS

The study protocol was approved by the Ethics Committee, and all patients signed a written informed consent form to have his information disclosed in this report. From February 2019 to November 2023, a total of 55 skeletally immature patients were identified with RPD who underwent either robot‐assisted or non‐robot‐assisted physeal‐sparing MPFL reconstruction. Patients in this retrospective case series were identified through a search of the institutional electronic medical records system. The status of the growth plate, lower extremity alignment, tibial tubercle‐trochlear groove (TT‐TG) distance (measured using CT), *Q*‐angle and femoral neck anteversion were assessed using pre‐operative radiographs, CT scans, and MRI. The operation duration, the number of fluoroscopies and guide needle punctures were recorded in both groups. The initial records search identified 98 patients who underwent physeal‐sparing MPFL reconstruction during the study period. Exclusion criteria were: previous knee surgery (*n* = 7), revision surgery (*n* = 2), TT‐TG > 22 mm (*n* = 14), genu valgum >10° (*n* = 4); femoral neck anteversion >25° (*n* = 6), Dejour Type D (*n* = 6) and severe rotation of the affected lower extremity >30° (*n* = 4). The anterior and distal tilt angles of the bone tunnel, as well as the distance between Schöttle point and the femoral insertion of the bone tunnel (DST), were recorded using post‐operative CT imaging in both groups [19]. Preoperative and postoperative International Knee Documentation Committee (IKDC), Kujala and Lysholm scores, and imaging evaluations such as standing long‐leg radiographs and patellar tilt angle (PTA) were used to assess the clinical outcomes.

### Robotic group: Robot‐assisted physeal‐sparing MPFL reconstruction

**Figure 1:**
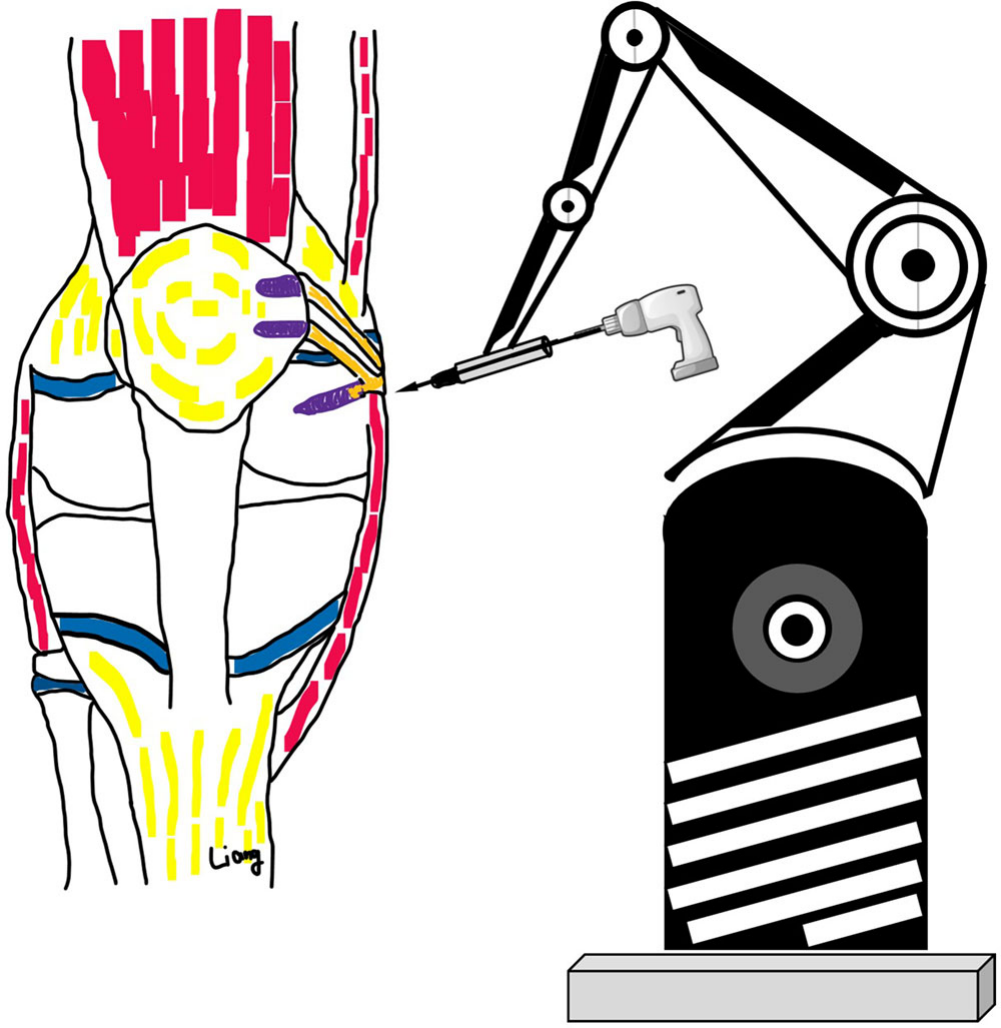
Schematic diagram of the preoperative plan for physeal‐sparing robot‐assisted MPFL anatomical reconstruction.

**Figure 2:**
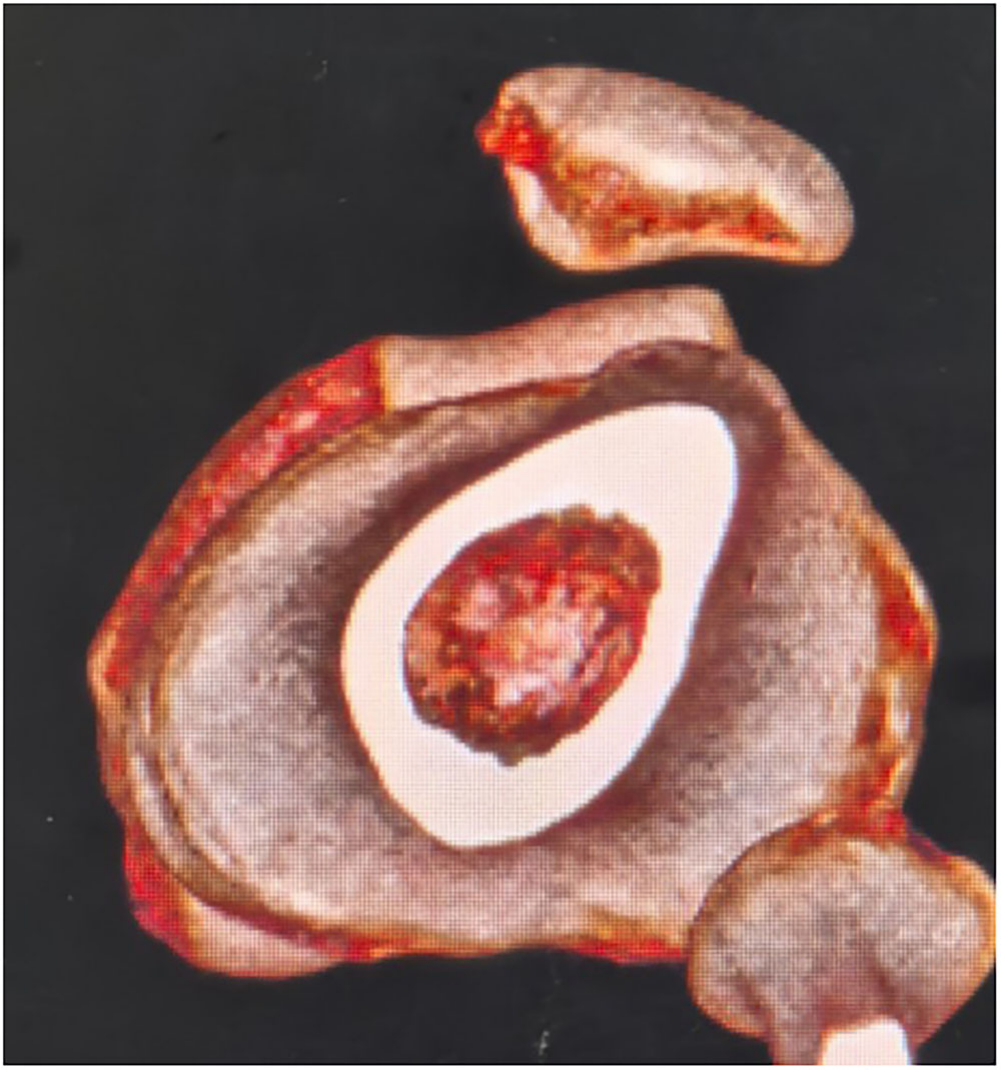
Preoperative 3D CT revealed patellar subluxation. 3D, three‐dimensional; CT, computed tomography.

**Figure 3:**
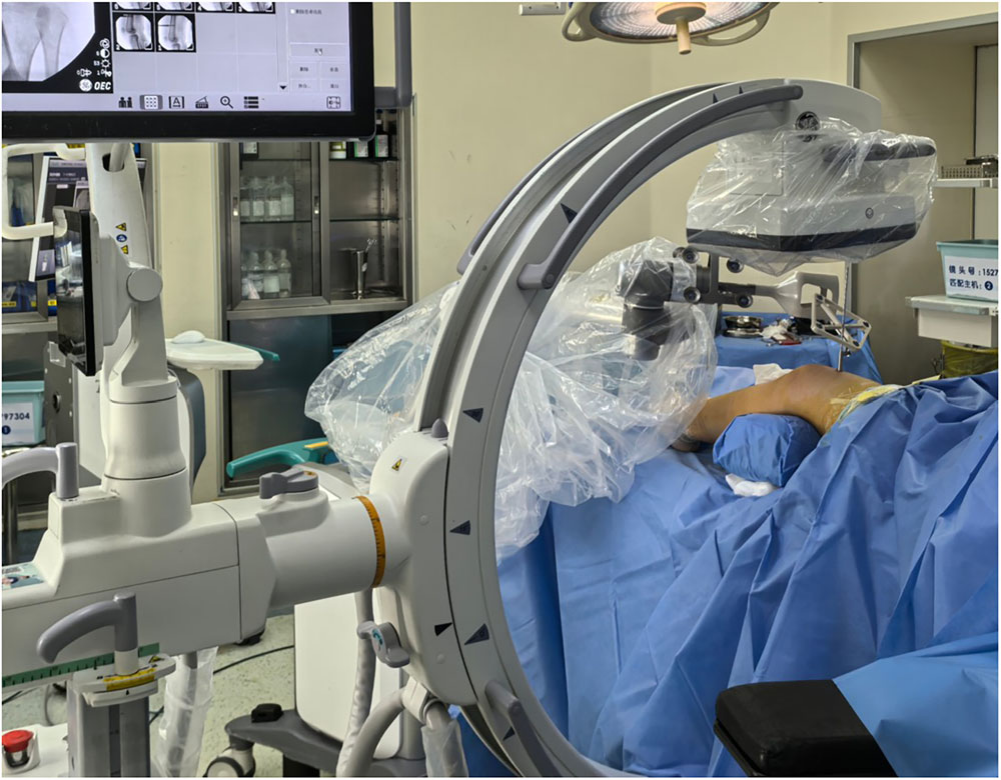
Intra‐operative 2D C‐arm, robot arm, tracker and workstation. 2D, two‐dimensional.

**Figure 4:**
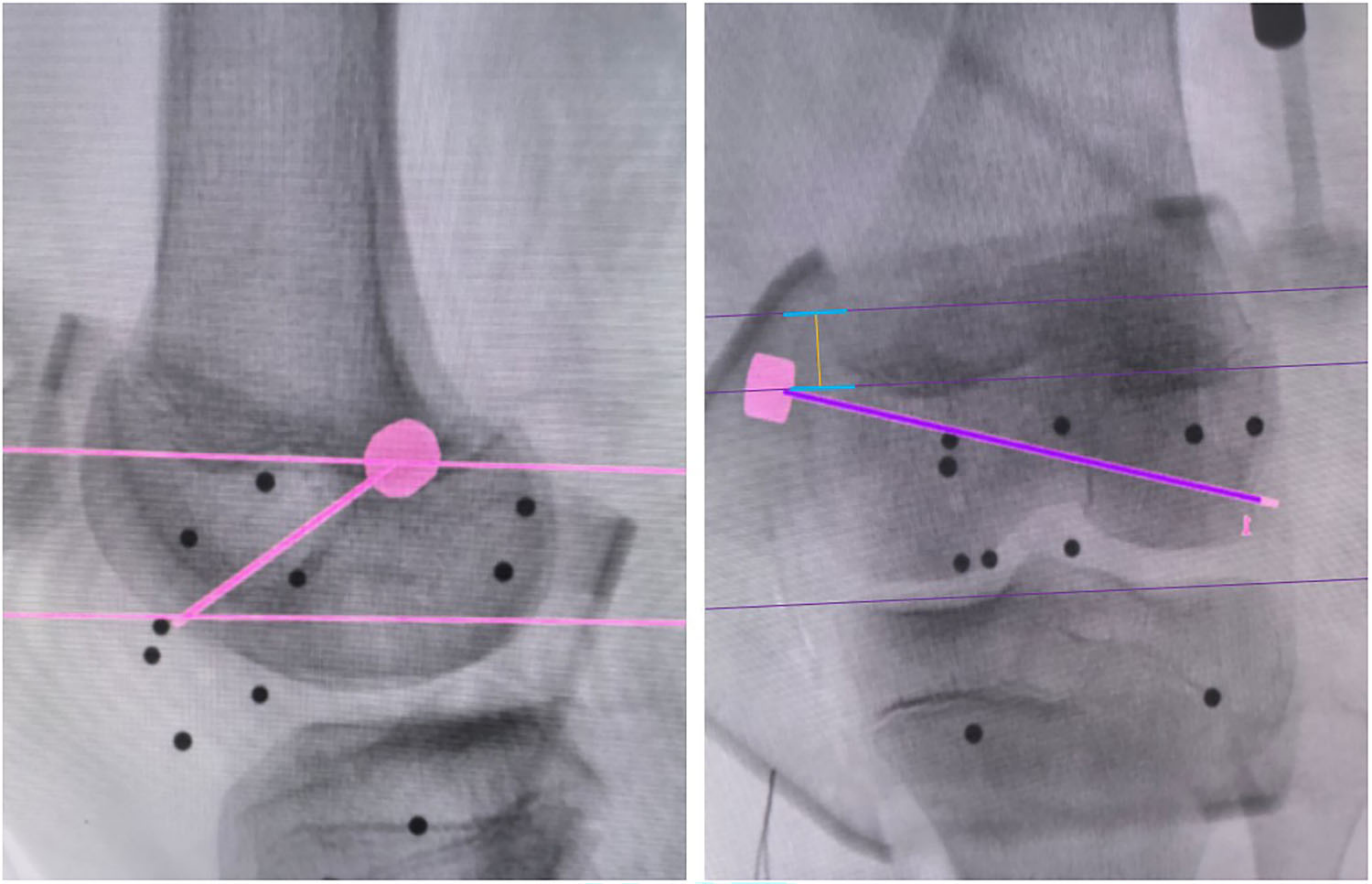
Intraoperative bone tunnel planning and measurements of the vertical distance (yellow line) between Schöttle point and the medial femoral physis.

**Figure 5:**
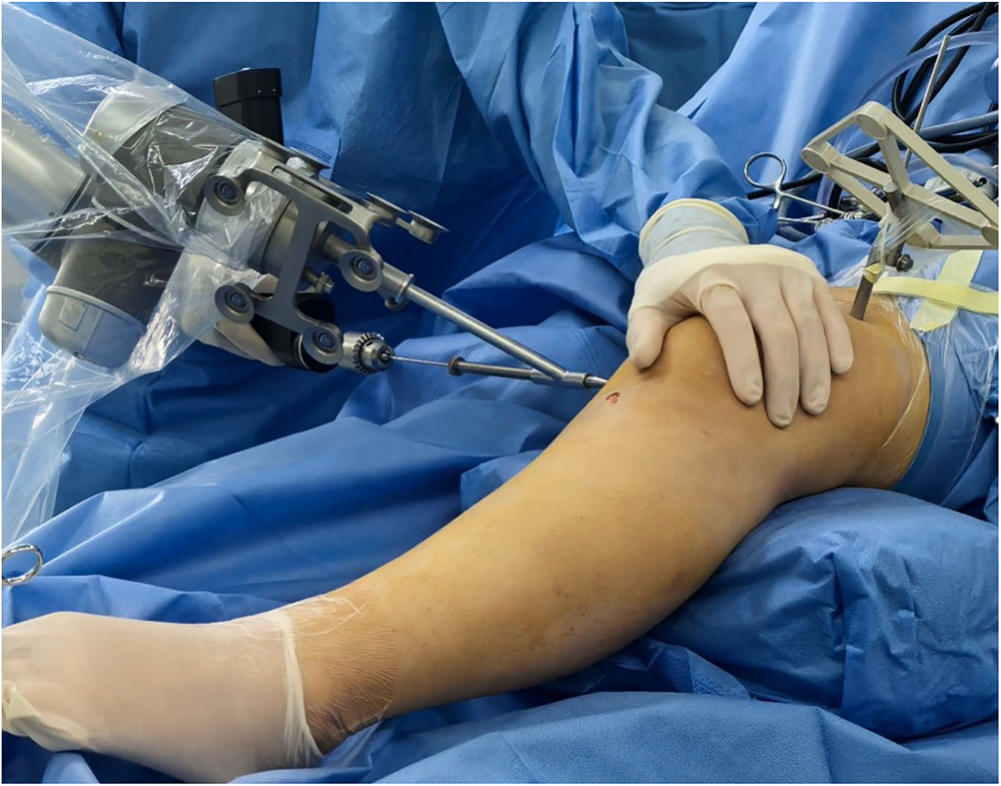
Robot‐guided placement of the surgical guide pin.

**Figure 6:**
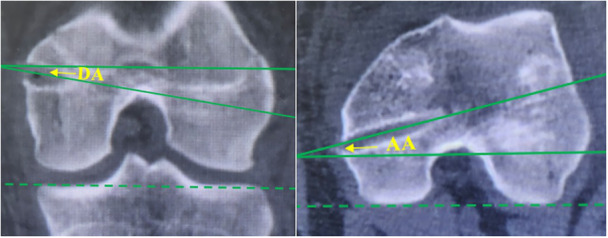
Measurement of DA and AA using post‐operative CT. AA, anterior tilt angle of the bone tunnel; CT, computed tomography; DA, distal tilt angle of the bone tunnel.

**Figure 7:**
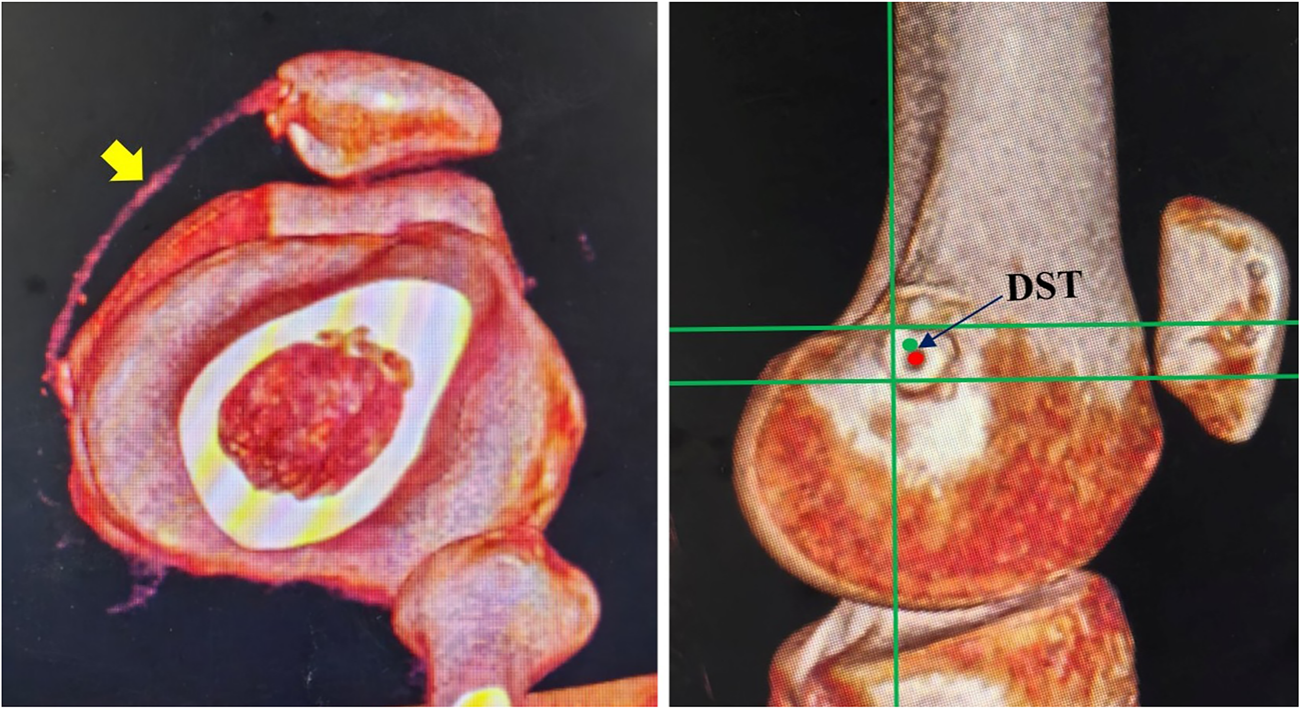
Post‐operative CT reconstruction revealed patella reduction, a reconstructed MPFL (yellow arrow) and DST measurement (The green dot indicates the Schöttle point, while the red dot indicates the actual femoral insertion.). CT, computed tomography; DST, the distance between the Schöttle point and femoral tunnel insertion; MPFL, medial patellofemoral ligament.

**Figure 8:**
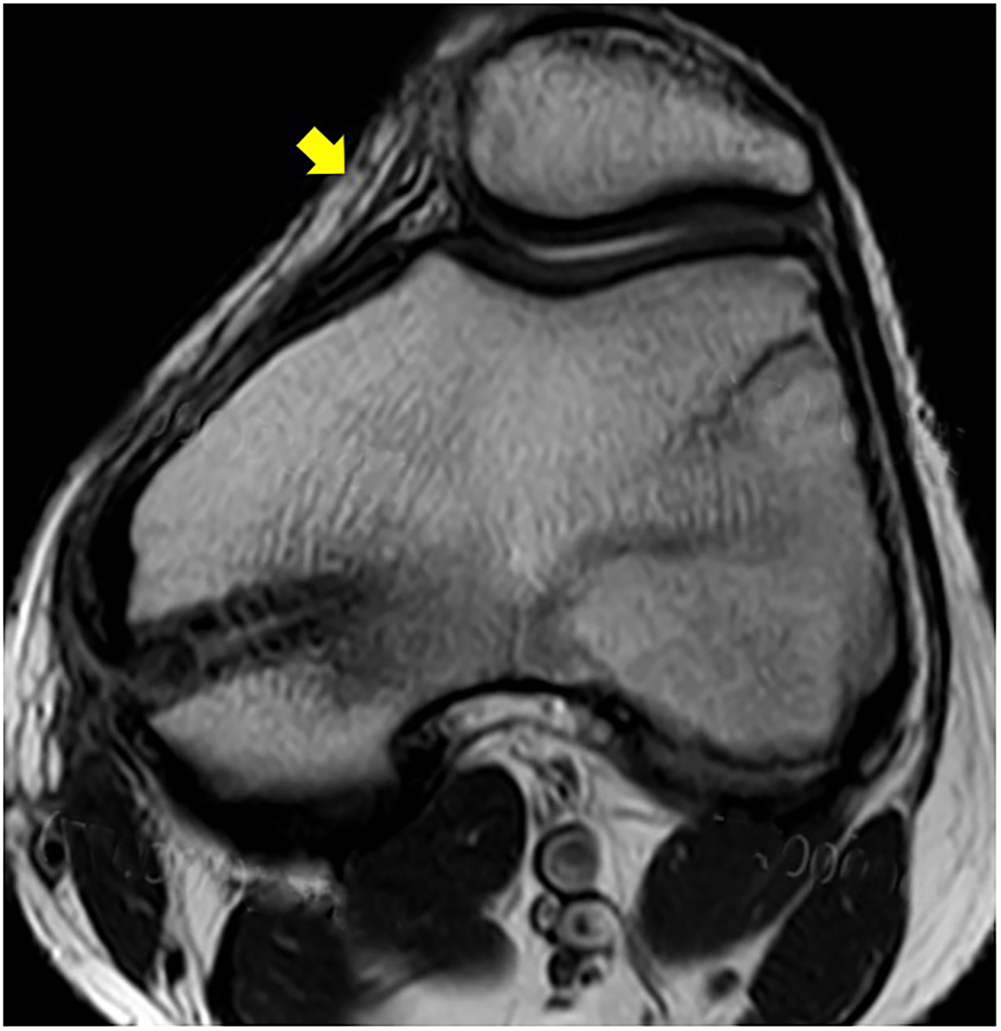
The MRI conducted 35 months after surgery showed a well‐preserved patellofemoral joint and the MPFL (yellow arrow). MPFL, medial patellofemoral ligament; MRI, magnetic resonance imaging.

**Figure 9:**
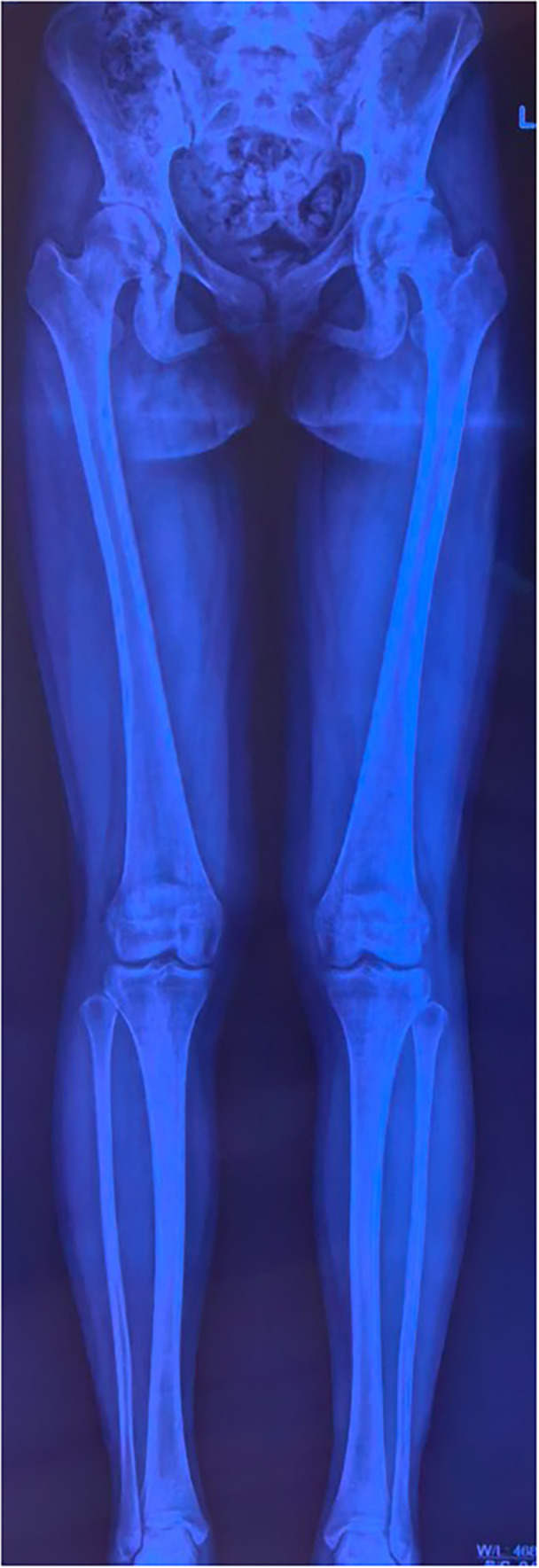
A standing long‐leg radiograph performed 40 months after surgery indicated good lower extremity alignment.

After general anaesthesia, the patient was positioned supine, and the semitendinosus tendon was prepared for use (Figure 1). A bone groove approximately 2 cm long and 2 mm deep was created along the medial margin of the patella. Two PEEK anchors (Smith & Nephew Inc.) were then implanted in the bone groove: one was placed in the middle of the upper third of the patella, and the other was positioned in the middle of the patella. The tracer from the surgical robot Tirobot® (TINAVI Medical Technologies Co., Ltd) was placed after a guide needle was put into the distal femur. The C‐arm (Perlove Medical Co., Ltd) was used to take intraopertive images, which were then transferred to the robotic workstation. The Schöttle point [16] was initially identified on the true lateral radiograph. A bone tunnel was then created in the distal epiphysis, extending from the Schöttle point to the lateral wall of the femoral lateral condyle. This bone tunnel trajectory was designed to avoid the physis, intercondylar notch, and the distal femoral cartilage, as determined using the image analysis system on the workstation. Meanwhile, the vertical distance between Schöttle point and the distal femoral physis was also measured using the system (Figure 4). Then, the optimal trajectory was chosen to prepare the bone tunnel. Meanwhile, the optical tracking device monitored the movement path and indicated any deviations. If the deviation exceeded 0.3 mm, the system would stop functioning and recommend a readjustment. The bone tunnel was then created using a 6‐mm reamer. After determining the isometric length of the ligament, the semitendinosus tendon was secured using an interference screw (Smith & Nephew Inc.). After the MPFL was reconstructed, arthroscopy was performed to check for excessive tightness, especially after knee flexion. The lateral retinaculum may be released if required. A typical case is shown in Figures 1, 2, 3, 4, 5, 6, 7, 8, 9.

### Intraoperative determination of the optimal bone tunnel path

**Figure 10:**
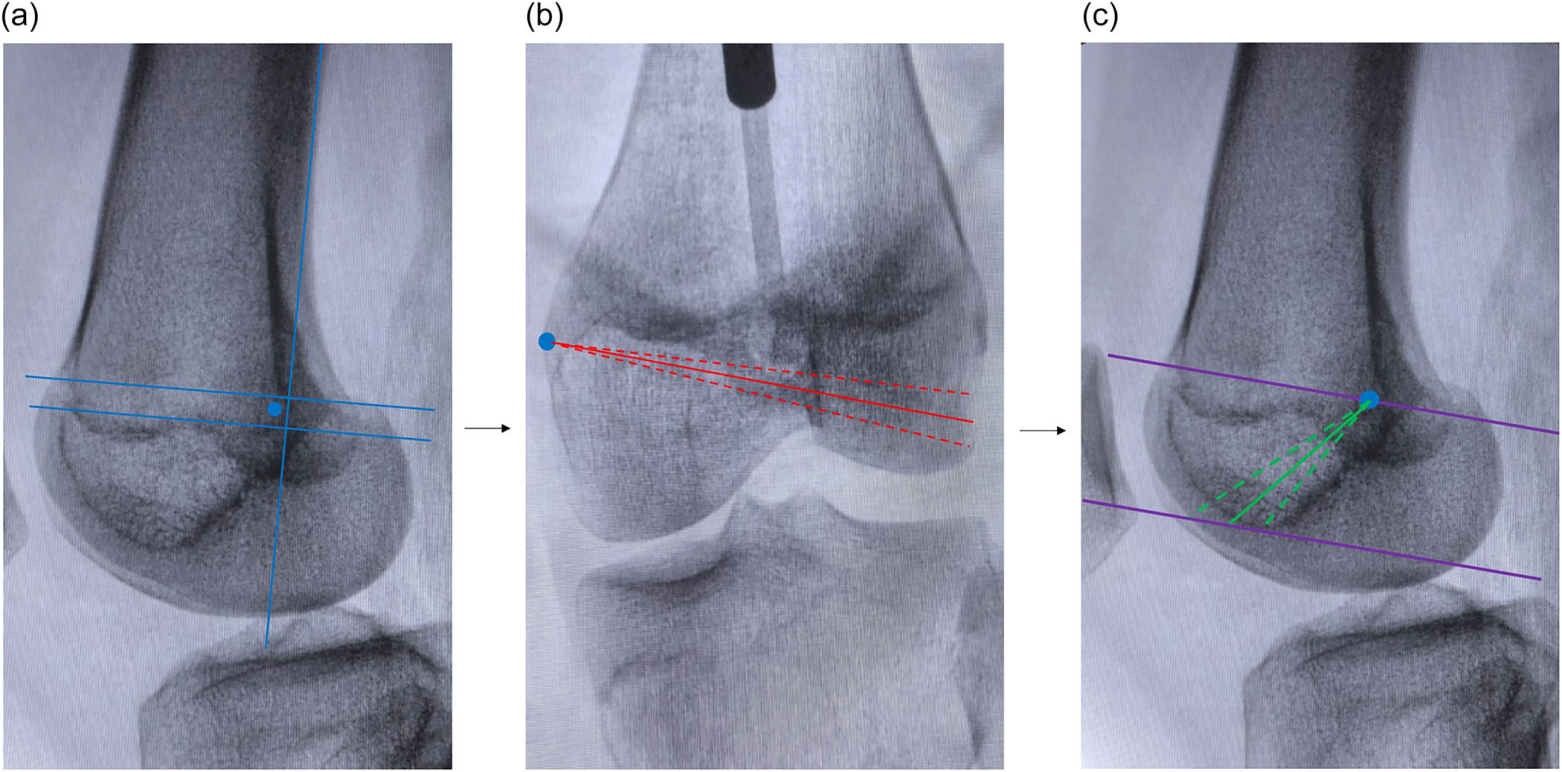
Schematic diagram of the optimal bone tunnel path planning. (a) Determination of the Schöttle point (blue dot) using lateral view. (b) Based on the Schöttle point, determine the extent of the bone tunnel path (red dashed line) and the optimal bone tunnel path (solid red line), which is positioned in the middle of the two red dashed lines. (c) After determining the optimal bone tunnel trajectory on the anteroposterior radiograph, the final optimal bone tunnel path (solid green line) is positioned in the middle of the two green dashed lines, with the permissible adjustment range (purple line).

During the operation, a 2D imaging navigation simulation system was employed to identify the optimal bone tunnel that would not affect the physis, intercondylar fossa, or the cartilage surface of the lateral femoral condyle. The schematic diagram is shown in Figure 10.

### Non‐robot‐assisted group

After general anaesthesia, the patient was positioned supine. The arthroscopic procedure was similar to that of the robot‐assisted group, but a freehand positioning fluoroscopy method was used for planning the femoral tunnel. First, the surgeon performed standard lateral fluoroscopy to identify the Schöttle point and then inserted a guide needle into the epiphysis at that location. Anteroposterior fluoroscopy was conducted to adjust the direction, ensuring that the procedure did not affect the physis, intercondylar fossa, or the cartilage surface of the lateral femoral condyle. After achieving satisfactory positioning, the preparation of the bone tunnel and subsequent operations followed the same protocol as those of the robot‐assisted group.

### Statistical analysis

Statistical analysis was performed using SPSS 20.0 software (IBM). The normality of measurement data was examined. These data are presented as mean ± standard deviation (*x* ± SD) and range. Comparisons between groups were conducted using independent samples *t* tests. Categorical data (such as sex) are presented as frequency (*n*, %), and comparisons between groups were performed using chi‐square tests. A statistically significant difference was defined as *p* < 0.05.

## RESULTS

The details of the patients were presented in Table 1. The mean age of the study was 13.1 ± 1.5 years (range, 11–16 years). All patients returned for follow‐up at a mean of 35.5 ± 8.5 (range, 16–57) months after surgery, with no significant difference between the two groups (36.7 ± 8.8 vs. 34.1 ± 8.3, *p* = 0.275). The mean distance from the Schöttle point to medial femoral physis was 6.97 ± 1.92 mm, measured with a 2D intraoperative navigation system in the robot‐assisted group, and all Schöttle points were located below the physis in every patient. The distal and anterior tilt angles of the bone tunnel, the distance between the Schöttle point and the femoral tunnel insertion (DST), as well as the intraoperative parameters, are illustrated in Table 2. No patients reported recurrent patellar instability after surgery. Normal patellar tracking was observed post‐operatively in all study patients and significantly improved functional scores, and patellar tilt angles were noted at the final follow‐up (*p* < 0.05). The IKDC, Lysholm and Kujala scores in the robot‐assisted group were significantly higher than those in the non‐robot‐assisted group three months post‐operatively; however, the knee function scores and patellar tilt angles at the last follow‐up showed no significant differences between the two groups (Table 3). MRIs (T1‐weighted and T2‐weighted images) performed 1 year post‐operatively did not reveal any signs of dysfunction in the reconstructed MPFL or osteochondral injury. No instances of lower extremity malalignment or physeal invasion were observed in the robot‐assisted group.

**Table 1:** Baseline patient characteristics.

Demographic factor	Robotic group	Non‐robotic group	*p*
Number of cases	30	25	‐
Sex (male/female)	12 (40.0%)/18 (60.0%)	11 (44.0%)/14 (56.0%)	0.765
Age (years)	12.9 ± 1.5 (11–16)	13.3 ± 1.6 (11–16)	0.325
IKDC score	29.0 ± 5.2 (15–39)	27.1 ± 4.3 (16–35)	0.144
Lysholm score	31.3 ± 7.6 (19–44)	30.7 ± 6.8 (18–46)	0.771
Kujala score	27.1 ± 5.0 (17–36)	24.8 ± 4.7 (16–34)	0.089
Patellar tilt angle (°)	27.9 ± 5.2 (16–37)	27.0 ± 6.1 (19–40)	0.575
TT‐TG (mm)	18.3 ± 2.2 (13.4–21.9)	17.9 ± 2.5 (12.6–21.2)	0.515
Q‐angle (°)	14.2 ± 2.4 (9.6–17.7)	14.6 ± 2.6 (10.7–18.0)	0.531
Beighton score	3.0 ± 2.2 (0–7)	3.6 ± 2.1 (0–7)	0.335

*Note*: Results are presented as *n* (%) or mean ± SD (range).

Abbreviations: IKDC, International Knee Documentation Committee; TT‐TG, tibial tubercle‐trochlear groove.

**Table 2:** The intraoperative and post‐operative observation parameters.

Parameter	Robotic group	Non‐robotic group	*p*
Operation duration (min)	90.1 ± 12.3 (68–115)	84.0 ± 15.1 (63–106)	0.096
Fluoroscopy number (times)	3.7 ± 0.5 (3–5)	10.3 ± 1.8 (8–14)	<0.001
Number of guide needle punctures (times)	1.1 ± 0.3 (1–2)	5.7 ± 1.1 (4–8)	<0.001
DST (mm)	2.00 ± 0.84	5.45 ± 1.74	<0.001
DA (°)	13.8 ± 1.7 (10.6–17.4)	11.4 ± 1.5 (9.8–15.3)	<0.001
AA (°)	14.5 ± 1.7 (11.1–18.9)	16.6 ± 4.7 (9.7–24.0)	0.044

Abbreviations: AA, anterior tilt angle of the bone tunnel; DA, distal tilt angle of the bone tunnel; DST, the distance between the Schöttle point and femoral tunnel insertion.

**Table 3:** Comparison of post‐operative follow‐up results.

Time	Parameter	Robotic group	Non‐robotic group	*p*
At 3 months post‐operatively	IKDC score	87.1 ± 6.1 (73–96)*	82.9 ± 5.7 (70–92)*	0.011
	Lysholm score	85.3 ± 5.7 (71–93)*	81.1 ± 5.2 (73–93)*	0.007
	Kujala score	82.7 ± 6.0 (69–90)*	77.5 ± 5.1 (70–87)*	0.001
At the last follow‐up	IKDC score	93.3 ± 5.6 (79–100)*	90.4 ± 5.7 (81–98)*	0.062
	Lysholm score	91.6 ± 6.1 (75–99)*	88.6 ± 6.3 (77–98)*	0.079
	Kujala score	89.2 ± 6.2 (74–97)*	86.1 ± 5.9 (75–94)*	0.062
	PTA (°)	13.8 ± 3.3 (8.7–22.3)*	13.8 ± 3.3 (9.5–21.5)*	0.411

* *p* < 0.05, Compared with the preoperative values presented in Table 1.

Abbreviations: IKDC, International Knee Documentation Committee; PTA, patellar tilt angle.

## DISCUSSION

The main finding of this study was that an anterodistal oblique bone tunnel can be safely utilized for MPFL anatomical reconstruction in skeletally immature patients, and robot‐assisted method is more accurate than freehand method, with fewer intra‐operative fluoroscopies and faster early recovery.

The inaccuracy of the femoral insertion is the primary risk for the failure of MPFL reconstruction. Furthermore, it is essential to consider the anatomical relationship between the MPFL insertion and the distal femoral physis in patients with skelatal immaturity [14, 15]. Despite the fact that many studies have decumented the relationship between the MPFL insertion and the physis, this relationship has not been precisely described, and no agreement has been established [11]. According to the findings of an indirect radiographic study by Shea et al. [17], the insertion of the MPFL was located 2–5 mm proximal to the distal femoral physis. Nonetheless, the majortiy of the references state the MPFL origin footprint is situated at or beneath the physis [2, 8, 18], which is consistent with our findings. In contrast to their findings, the direct intraoperative 2D navigation system utilized in this study may facilitate a more individualized anatomical MPFL reconstruction.

The Schöttle point was chosen for this study because it has been reported that all femoral insertion sites are located within a 7‐mm radius around the centre of the Schöttle point [9]. However, the relationships between the Schöttle point and the physis on anteroposterior radiographs may be deceptive when figuring out the relationship on lateral radiographs due to the concave curvature of the distal femoral physis [12]. In this study, the robot‐assisted method was used to position the femoral tunnel, aiming to be as close to the Schöttle point as possible. Consequently, post‐operative three‐dimensional CT reconstruction indicated a shorter DST in the robot‐assisted group.

In addition to the MPFL footprint, the orientation of the bone tunnel is significantly more important for protecting the physis. Some authors reported that drilling at angles of appropriately 15°–20° both distally and anteriorly minimizes damage to the physis, intercondylar notch and condylar cartilage [13]. In contrast, Irarrázaval et al. [7] reported much larger angles, measuring 30°–40° distally and 5°–35° anteriorly. Based on the results of the navigation system in this study, the closer the Schöttle point is to the physis, the larger the distal angulation required to avoid damaging the physis. Furthermore, in the non‐robot‐assisted group, the DST is larger, leading to a smaller distal tilt angulation. This smaller distal angulation allows for a larger cross‐sectional area within the epiphysis, which explains why the range of the anterior tilt angle is greater in the non‐robotic group. Moreover, the SD of the angles in the robotic group is smaller, indicating that optimal bone tunnel planning can achieve a relatively consistent angle. In contrast, the variation range of this angle in the non‐robot‐assisted group is too large, which increases the risk of iatrogenic injuries to the epiphysis, intercondylar notch, and cartilage surface. More importantly, these angles are likely to vary for each child; therefore, it may be beneficial for the robot to plan the position and orientation in advance.

### Advantages of robot‐assisted physeal‐sparing MPFL reconstruction

The present study demonstrated several benefits of utilizing robot‐assisted technology for MPFL reconstruction in skeletally immature patients. First, although Schöttle point was distal to the physis in all patients in this study, this does not imply that the angle of the screw can be arbitrarily placed at the distal epiphysis, as the diameter and orientation of the bone tunnel must also be considered. The robot can assist in one‐time positioning of the Schöttle point, planning the direction and diameter of the bone tunnel, ensuring that the coronal plane and cross‐section are positioned away from important anatomical structures, ultimately achieving an optimal bone tunnel angle that is close to perfect. Second, the robot was utilized for its ability to provide precise navigation and real‐time monitoring with an error margin of less than 0.5 mm [21], thereby ensuring a higher accuracy of implantation compared to non‐robot‐assisted procedures. In contrast, to achieve a relatively ideal insertion and angle, the non‐robot‐assisted group requires repeated punctures and fluoroscopy, which increases the degree of trauma imperceptibly. This may contribute to the lower functional scores in the early post‐operative period. Additionally, patients undergoing robotic surgery are informed that they are receiving a relatively safe and advanced procedure, which may help them quickly build confidence in their recovery after surgery.

## LIMITATIONS

Robotic assisted surgical system may be cost‐prohibitive for many institutions. In addition, robot‐assisted surgery may initially take a long time, but as technology improves, this duration is expected to decrease gradually. Longer‐term, prospective, randomized clinical studies would be helpful to further assess the clinical outcomes of this surgical technique.

## CONCLUSION

The intraoperative 2D robotic navigation system verified that the Schöttle point may be securely positioned in skeletally immature patients. The results of the study indicate that, in patients with unclosed physis, robot‐assisted design of anatomic reconstruction of the MPFL utilizing an anterodistal oblique bone tunnel is substantially safer and more accurate, showing positive short‐term clinical outcomes. We therefore propose that this surgical technique may be beneficial for skeletally immature patients with RPD.

## AUTHOR CONTRIBUTIONS

Qiuzhen Liang, Liang Zhang and Jiang Zheng conceptualized this study and performed the documentation. Zandong Zhao, Hongwei Zhan, Peidong Liu, Chaofan Liao, Junxuran Li, Yongchao Duan, Xin Kang, Bin Tian and Bo Ren performed data collection and analysis. All authors commented on the first draft of the manuscript and approved the final draft.

## CONFLICT OF INTEREST STATEMENT

The authors declare no conflicts of interest.

## ETHICS STATEMENT

The study protocol was approved by the Ethics Committee, and all patients signed a written informed consent form to have their information disclosed in this report.

## Data Availability

The data that support the findings of this study are available from the corresponding author upon reasonable request.
